# Overexpression of CA1 mRNA and the CA I Protein in Tumor Cells Does Not Change the Gene Expression of the ECM Proteins

**DOI:** 10.3390/ijms21020639

**Published:** 2020-01-18

**Authors:** Ján Lakota, Mária Dubrovčáková

**Affiliations:** 1 Biomedical Research Center, SAS, Dubravska cesta 9, 845 05 Bratislava, Slovakia; 2 Centre of Experimental Medicine, SAS, Dubravska cesta 9, 841 04 Bratislava, Slovakia

**Keywords:** carbonic anhydrase I, CA1 gene overexpression, tumor cell

## Abstract

In our study, we performed retroviral transduction to overexpress codon-optimized variant of gene encoding human carbonic anhydrase I (optiCA1) in two tumor cell lines PC3 and MDA-MB-231, derived from human prostatic and breast carcinoma respectively. We achieved significantly enhanced and stable overexpression of exogenous optiCA1 gene. The expression of endogenous, wild CA1 gene was found to be normally low (C_t_ 28.6 for PC3 cells) or below to the detection limit (C_t_ 35.5 for MDA-MB-231 cells). No morphological changes and no decreasing viability of tumor cells were observed upon stable overexpression of the optiCA1 gene. In our study we have shown that the overexpression of the optimized human CA1 in engineered PC3 and MDA-MB-231 cells did not induce similar changes as we observed in tumor cells cultivated in the presence of human sera containing extensively high titers of anti-CA I autoantibodies from patients with complete remission of malignant disease. In both optiCA1transduced cell lines, the expression of selected genes responsible for basal lamina assembly, cytoskeleton, extracellular matrix proteins and proto-oncogenes (COL1A1, COL4A4, LAMC2, CTHRC1, and WNT7B) was not changed.

## 1. Introduction

Carbonic anhydrase I (CA I) together with other 14 members of family of carbonic anhydrases, catalyzes the reversible hydration of carbon dioxide [1,2]. The CA I enzyme is abundant in erythrocytes (CA I is the second most abundant protein after hemoglobin), but its catalytic efficiency is relatively low [3]. However, the familiar homozygous deficiency of carbonic anhydrase I with virtually absent CA I has no clinical consequences with no hematological and renal defects [4]. The presence of high levels of autoantibodies against CA I (anti-CA I Abs) in the patients’ sera was found to be linked with spontaneous tumor regression and improved patient survival, but also with the suppression of hematopoiesis [5,6]. The spectrum of the spontaneously regessed tumors are wide: Hodgkin’s disease, non-Hodgkin lymphoma, Ewings’s sarcoma and breast cancer [6], multiple myeloma and ovarian cancer (data not shown). Tumor cells of different origin were cultivated in vitro in the media supplemented with patients’ sera positive for anti-CA I Abs. As stated previously in the RNA microarray report: “The downregulated genes in cells treated in the presence of patients’ sera positive for anti-CA I Abs compared to the negative sera include several genes related to adhesion and cytoskeleton, such as different collagens and keratin family members. In addition, some proto-oncogene WNT7B was downregulated as well” [7]. More precisely, these cells displayed decreased expression of mRNAs of the genes associated with extracellular matrix (ECM) and basal lamina (BL) assembly (COL1A1–collagen I, COL4A4–collagen IV, LAMC2–laminin gamma), cytoskeleton (KRT14–keratin 14 type I), WNT7B and collagen triple helix repeat containing 1 (CTHRC1). Surprisingly, the expression of mRNA of the CA1 gene was increased (up to thirteen times in the PC3 tumor cell line) [7]. This observation led us to examine the possible association of the CA I with the overexpression on the abovementioned “non CA1 genes”. Therefore, we have performed retroviral transduction to constantly overexpress codon-optimized variant of gene encoding CA I (optiCA1) in PC3 and MDA-MB-231 tumor cells. We were not able to see any association of the increased CA1 expression (tested with the codon-optimized variant of gene encoding human carbonic anhydrase I) with the decreased mRNA expression of genes associated with the ECM and BL assembly and the WNT7B and CTHRC.

## 2. Results

### 2.1. Overexpression of Codon Optimised Human CA1 Gene Reduces Endogenous CA1 Gene Expression but Does Not Change Expression of Genes Encoding Extracellular Matrix Proteins

**Figure 1:**
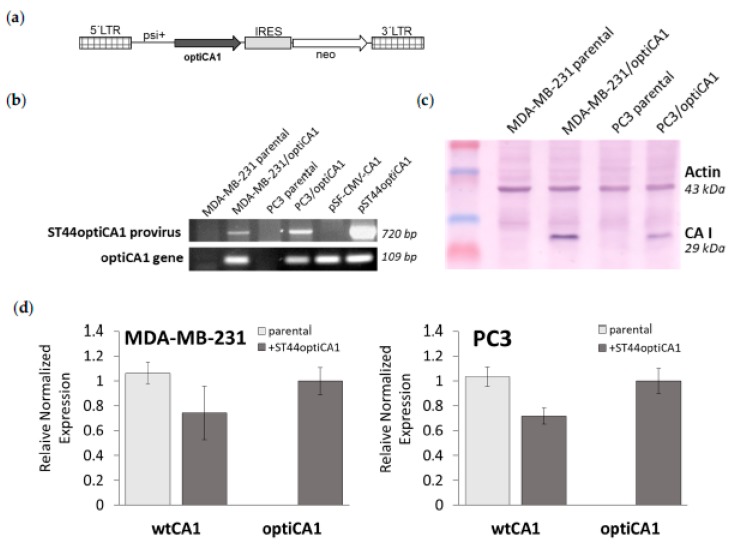
Genetically engineered tumor cells MDA-MB-231 and PC3 stable overexpress codon optimized human CA1 gene (optiCA1). (**a**) Structure of provirus ST44optiCA1. LTR, long terminal repeat; IRES, internal ribosomal entry site; psi+, packaging signal; neo, neomycin resistance gene; optiCA1, optiCA1 gene subcloned from pSF-CMV-CA1 plasmid (Oxford Genetics). (**b**) Detection of integrated provirus ST44optiCA1 in genomic DNA of tumor cells by PCR; vector specific psi+ sense primer and optiCA1 specific antisense primer optiCA1 rev for detection of provirus; primers optiCA1 for and optiCA1 rev for detection of optiCA1 gene overexpression; plasmids DNA pSF-CMV-CA1 and pST44optiCA1 were used as positive controls. (**c**) Western blot analysis of overexpressed protein CA I in engineered tumor cells MDA-MB-231/optiCA1 and PC3/optiCA1 detected by monoclonal anti-human CA I antibody (anti-CAI); anti-Actin, polyclonal anti-human Actin antibody. (**d**) Reverse transcriptase quantitative PCR; The mRNA expression of optiCA1 transgene driven from integrated ST44optiCA1 provirus and endogenous native CA1 gene (endoCA1); the data are expressed as means of quadruplicates ± SD.

The optiCA1 version of human CA1 gene was excised from commercial expression vector pSF-CMV-CA1 (Origene) and subcloned into the retroviral vector pJZ308 as described in [8], creating ST44optiCA1 retrovirus (Figure 1a). Because not all tRNAs are expressed equally, codon optimization of CA1 DNA sequence in pSF-CMV-CA1 by changing its codons was made to match the most prevalent tRNAs. This enables more efficient translation of target CA1 gene. Alignment of DNA of codon optimized version (optiCA1) and endogenous native (endoCA1) gene revealed identity of 75%, while amino acid sequences of CA1 remained 100% identical. Transduction using retrovirus ST44optiCA1 guaranteed stable overexpression of codon optimized human optiCA1 gene in prostatic tumor cells PC3 (PC3/optiCA1) and breast carcinoma cells MDA-MB-231 (MDA-MB-231/optiCA1) (Figure 1b). Overexpression of CA1 gene was confirmed by Western blot (Figure 1c), but concurrently, the expression of endoCA1 native gene was found to be decreased at the level of 70–80% (Figure 1d).

**Figure 2:**
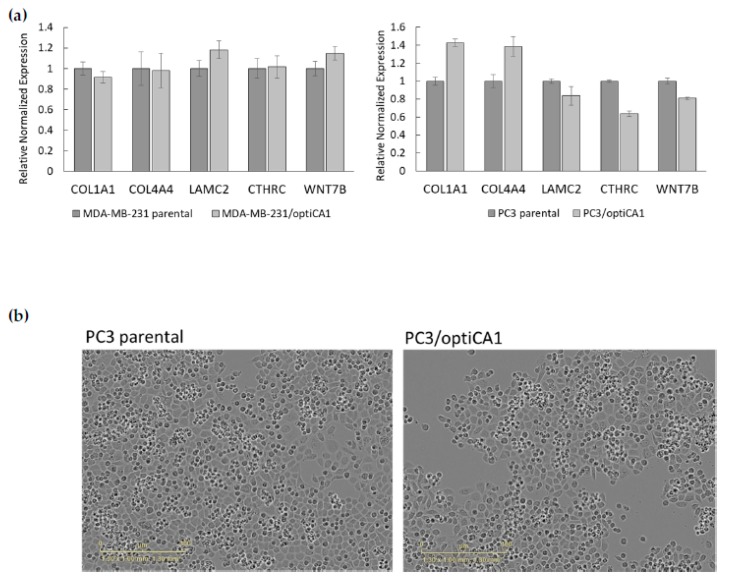
Expression of selected genes responsible for adhesion, cytoskeleton and selected proto-oncogenes and cellular morphology are not changed in PC3 prostatic tumor cells engineered to stable overexpress codon optimized CA1 gene (optiCA1; PC3/optiCA1). (**a**) Reverse transcriptase quantitative PCR; COL1A1–alpha-1 type I collagen, COL4A4–alpha-4 type IV collagen, LAMC2–laminin subunit gamma-2, CTHRC1 – collagen triple helix repeat containing protein 1, WNT7B–Wingless-Type MMTV Integration Site Family, Member 7B; the data are expressed as means of quadruplicates ± SD. (**b**) morphology of parental PC3 and PC3/optiCA1 cells; light-microscope images from IncuCyte ZOOM Kinetic Imaging System, scale bar: 300 μm.

The expression of selected genes encoding extracellular matrix and basal lamina components (COL1A1–collagen I, COL4A4–collagen IV, LAMC2–laminin gamma), and proto-oncogenes (CTHRC1–collagen triple helix repeat containing protein 1, WNT7B–Wingless-Type MMTV Integration Site Family, Member 7) were not significantly changed in optiCA1 overexpressing tumor cells (Figure 2a). The cell morphology, which was linked with altered expression profile as we reported previously [7], was not changed when optiCA1 was overexpressed (Figure 2b).

### 2.2. Overexpression of Codon Optimised Human CA1 Gene Doesn’t Reduce Short Term Cell Proliferation

**Figure 3:**
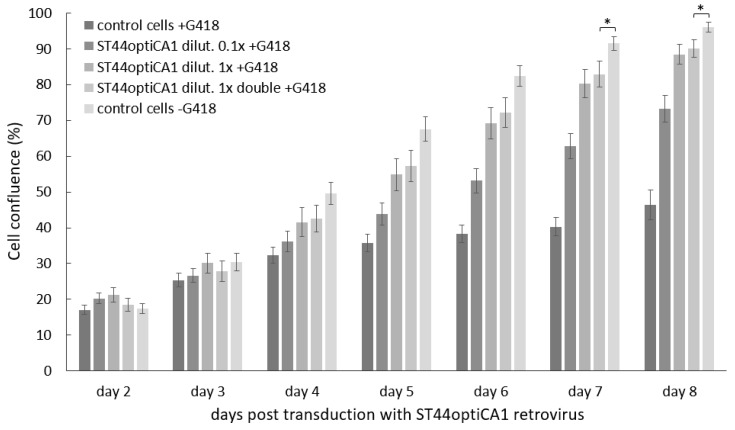
Proliferation of transduced tumor cells overexpressing optimized human CA1 gene from ST44optiCA1 provirus. Virus containing medium was used diluted 10-times (0.1×), undiluted (1×) in one transduction or undiluted in two subsequent transductions (1× double). G418–geneticin for selection of transduced cells; kinetic measurement of the cell proliferation is expressed as the cell confluence scanned by IncuCyte ZOOM Kinetic Imaging System; values are expressed as the means of 14-plicates; bars represent SEMs. * statistical significance (*p* < 0.05)

After transduction with ST44optiCA1 retrovirus, the growth of transduced tumor cells was not reduced rapidly in the first days (*p* = 0.02–0.05 on days 7 and 8). During selection with geneticin (G418), the cell confluence of 100 % was reached within 9 days (Figure 3) and transduced resistant optiCA1 tumor cells proliferated. Dilution of ST44optiCA1 virus containing media revealed successful transduction also with ten-times diluted media (dilution 0.1×; Figure 3), indicating high titer of the virus. We achieved multiplicity of infection (MOI) approx. of 5–10. After long term cultivation (up to 12 weeks), and after four passages, optiCA1 overexpressing PC3 cells were found to be significantly lagging in the growth and proliferation in comparison to the parental cells (data not shown). The viability of the cells was not affected.

## 3. Discussion

In the sera of cancer patients with spontaneous tumor regression after high dose therapy and autologous stem cell transplantation, a high titer of human anti-CA1 autoantibodies (anti-CA I Abs) was discovered [5]. In vitro, in the tumor cell line cultures, the anti-CA I-positive patients’ sera induced changes of the cell morphology and downregulated expression of selected genes responsible for the formation of BL, ECM, and cytoskeleton. The expression of proto-oncogenes WNT7B and CTHRC1 was decreased too [7]. Simultaneously, in the presence of sera with anti-CA I Abs, the expression of the CA1 gene mRNA was upregulated among all tested tumor cell lines. In prostatic tumor cells PC3, CA1 mRNA expression increased up to thirteen times during anti-CA I Abs-positive patient’s sera treatment. If the cells were grown in the presence of human sera negative for anti-CA I Abs the expression of endogenous CA1 mRNA remained rather low [7].

We were curious about the association between the upregulation of CA1 gene and the downregulation of the genes responsible for the formation of BL, ECM, and some proto-oncogenes (WNT7B and CTHRC). Therefore, we performed retroviral transduction to overexpress codon-optimized variant of gene encoding human carbonic anhydrase I (optiCA1) in two tumor cell lines PC3 and MDA-MB-231. Codon optimization is switching the codons used in a transgene without changing the amino acid sequence that it encodes for. This typically dramatically increases the abundance of the protein the codon optimized gene encodes because it removes “rare” codons and replaces them with abundant codons. So, in order to efficiently express protein in higher quantities, the more abundant of the degenerate tRNAs have to be used. Thus, a gene can be mutated (or synthetized de novo) to change the codons used for coding particular amino acids, without changing the amino acid sequence of the protein itself. Rare codons are replaced by codons that are more abundant in the genes of the host organism. Using transduction with retroviral vector, the overexpression of desired gene can be enhanced more than 100-times and remains stable for weeks [8]. With stable optiCA1 gene overexpression, we intended to achieve increased of CA1 levels analogous to those reported after cultivating anti-CA I Abs positive patients’ sera linked with favorable prognosis of malignant disease. However, we observed that optiCA1 overexpressing tumor cells, when compared with the controls, had not statistically changed expression profile of mRNAs (COL1A1, COL4A4, LAMC2, CTHRC1, and WNT7B). Nevertheless, we observed a slight (but not statistically significant) downregulation of the expression of innate endogenous CA1 gene in tumor cells. However, one should be aware although the amino acid sequence of the “opti” CA I is the same as in the wild type CA I enzyme the sequence of CA1 mRNA in the optiCA1 gene is differs from the wild type CA1 mRNA.

The gene encoding carbonic anhydrase I or carbonic anhydrase I protein in humans is not essential, as it has been shown in the study of familial deficiency of CA I synthesis [4]. The CA I role and exact mechanism of CA I action in spontaneous tumor regression remains perplexing. In our pilot study we have shown, that the optiCA1 overexpression alone (in the short-time period after transduction, i.e., up to 10 days) does not mimic the effect of the anti-CA I Abs-positive patients’ sera on tumor cells in vitro. The future tasks are further studies of the long-term period effect(s) of the optiCA1 overexpression on cellular processes and to prove the tumorigenicity of engineered optiCA1 overexpressing cells in vivo.

## 4. Materials and Methods

### 4.1. Cell Lines and Chemicals

Human breast adenocarcinoma cell line MDA-MB-231 (ECACC 92020424) and human prostate adenocarcinoma cell line derived from metastatic site PC3 (ATCCCRL-1435TM) were maintained in high-glucose (4.5 mg/mL) Dulbecco’s modified Eagle’s medium (DMEM; Biochrom AG, Berlin, Germany) supplemented with 10% fetal bovine serum (FS) (Biochrom AG, Berlin, Germany), and 2 mM glutamine. Retroviral packaging cell lines GP+E-86 (ATCC No. CRL-9642) and GP+envAM12 (ATCC No. CRL-9641; both kindly provided by Dr J. Bies, NCI NIH, Bethesda, MD, USA) derived from mouse fibroblast cell line were maintained in high-glucose DMEM supplemented with 5% FS. All cells were maintained in humidified atmosphere at 37 °C and 5% CO2.

All chemicals were purchased from Sigma-Aldrich (St. Louis, MO, USA) if not stated otherwise.

### 4.2. Construction of Recombinant Retroviral Vector Containing Human CA1 Gene and Retrovirus Production

Intronless human CA1 gene (codon optimized version; optiCA1) was cloned from the pSF-CMV-CA1 expression vector (Oxford Genetics Ltd., Oxford, UK) using standard cloning techniques into the bicistronic retroviral vector pJZ308 derived from Moloney murine leukemia virus (MoMuLV) [8]. The optiCA1 gene encodes identical protein sequence to the endogenous CA1 gene. Retroviral construct named pST44optiCA1 (Figure 1A) was verified by PCR and the open reading frame of the optiCA1 gene was sequenced. Retroviral vector pST44optiCA1 was used for production of replication deficient retroviral particles ST44optiCA1 in packaging cells GP+E-86 and GP+envAM12 as described in [8]. Virus-containing medium for transduction of target tumor cells was collected from semi-confluent cultures of GP+envAM12/ST44optiCA1, filtered through a 0.45-μm filter, and used either fresh or kept frozen at − 80 °C.

### 4.3. Western Blotting

The cell lysates were centrifuged (10 min at 17,000× *g*), and the total protein concentrations in supernatants were determined by BCA assay. Samples of 100 μg total proteins were separated using electrophoresis in the 10% SDS-PAGE and blotted onto the PVDF membrane (Millipore, Billerica, MA, USA). The membrane was blocked for 2 h in a blocking buffer containing 5% non-fat milk in PBS with 0.1% Tween 20. Incubation with primary antibodies was performed overnight at 4 °C. A CA I mouse monoclonal antibody (Moravian Biotechnology, Brno, Czech Republic) was diluted 1:1000 in 3% BSA in TBST. Then, the membrane was washed with 0.1% Tween 20 in PBS, incubated for 1 h (RT) with secondary antibody (Sigma-Aldrich, St. Louis, MO, USA), washed again, and developed with the ECL detection system [9].

### 4.4. Gene Expression Analysis

Reverse transcriptase quantitative PCR (RT-qPCR) was performed as previously described [7]. Briefly, RT-qPCR was performed using total RNA extracted from 5.0 × 10^5^ cultured cells using the NucleoSpin RNA II kit (Macherey-Nagel, Dueren, Germany). RNA was depleted from genomic DNA by DNase treatment (DNase I, RNase-free; Thermo Fisher Scientific, Waltham, MA, USA) and 1.15 µg of total RNA was reverse transcribed using the SensiFAST cDNA Synthesis kit (Bioline, London, UK). Quantitative RT-PCR was performed in quadruplicates in total volume 19 µL Brilliant III Ultra-Fast SYBR QPCR Master Mix (Agilent Technologies, Santa Clara, CA, USA), 0.25 pmol/L concentration of primers and 0.5 µL template cDNA per one reaction. The protocol for RT-qPCR was started with the activation step at 95 °C for 3 min. and followed by 45 cycles of the denaturation step at 95 °C for 15 sec. and annealing/polymerization at 60 °C for 15 s. with plate read steps at 75 °C and 80 °C. PCR was performed in Bio-Rad 96FX cycler (Bio-Rad Laboratories, Hercules, CA, USA). The analysis was done using Bio-Rad CFX Manager software version 1.6 as normalized fold expression using the $\Delta 2^{-\Delta \Delta Ct}$ method. The gene for hypoxanthine phosphoribosyltransferase 1 (HPRT1) was used as the reference gene. Primers for selected genes encoding proteins of extracellular matrix, basal lamina and proto-oncogenes were used as listed in [7]. For monitoring human optiCA1 gene expression after stable retroviral transduction with ST44otiCA1 retrovirus, primers optiCA1 for: 5′-GCTGATGAAGGTAGGCGAAG-3′ and optiCA1 rev: 5′-GGAAGGATCGAAATTCGTGA-3′ were used. For gene expression analysis of endogenous human CA1 gene, primers endoCA1 for: 5′-TAAAACCAAGGGCAAACGAG-3′ and endoCA1 rev: 5′-GGCTGTGTTCTTGAGGAAGG-3′ were used. Primers were designed using Primer3 Input software, version 0.4.0 (http://frodo.wi.mit.edu/primer3) and synthetized by Metabion, International (Martinsried, Germany).

### 4.5. Kinetic Measurement of Cell Proliferation

Tumor cells were seeded at density of 2.0 × 10^4^ cells per well in 6-well plates and left to start to grow. On the third day, ST44optiCA1 retrovirus containing medium was added to the dividing cells. After 24 h, selection was performed by adding geneticin (G418), a selection antibiotic to ensure the selection of transduced cells overexpressing optiCA1 gene. Cell confluence was monitored by IncuCyte ZOOM Kinetic Imaging System (Essen BioScience, Welwyn Garden City, UK). Phase contrast images were acquired every 24 h and 14 images were taken per well. Data were analyzed using IncuCyte ZOOM software. Proliferation was expressed as mean values of cell confluence in % ± SEM.
